# Mitochondria-Mediated Azole Drug Resistance and Fungal Pathogenicity: Opportunities for Therapeutic Development

**DOI:** 10.3390/microorganisms8101574

**Published:** 2020-10-13

**Authors:** Jinxing Song, Jingwen Zhou, Lei Zhang, Rongpeng Li

**Affiliations:** 1 The Key Laboratory of Biotechnology for Medicinal Plants of Jiangsu Province and School of Life Science, Jiangsu Normal University, Xuzhou, Jiangsu 221116, China; 3020182325@jsnu.edu.cn; 2 Shandong Provincial Key Laboratory of Infection and Immunity, Jinan 250012, China; leizhang@sdu.edu.cn

**Keywords:** azole resistance, pathogenic fungi, mitochondria, calcium signaling

## Abstract

In recent years, the role of mitochondria in pathogenic fungi in terms of azole resistance and fungal pathogenicity has been a rapidly developing field. In this review, we describe the molecular mechanisms by which mitochondria are involved in regulating azole resistance and fungal pathogenicity. Mitochondrial function is involved in the regulation of drug efflux pumps at the transcriptional and posttranslational levels. On the one hand, defects in mitochondrial function can serve as the signal leading to activation of calcium signaling and the pleiotropic drug resistance pathway and, therefore, can globally upregulate the expression of drug efflux pump genes, leading to azole drug resistance. On the other hand, mitochondria also contribute to azole resistance through modulation of drug efflux pump localization and activity. Mitochondria further contribute to azole resistance through participating in iron homeostasis and lipid biosynthesis. Additionally, mitochondrial dynamics play an important role in azole resistance. Meanwhile, mitochondrial morphology is important for fungal virulence, playing roles in growth in stressful conditions in a host. Furthermore, there is a close link between mitochondrial respiration and fungal virulence, and mitochondrial respiration plays an important role in morphogenetic transition, hypoxia adaptation, and cell wall biosynthesis. Finally, we discuss the possibility for targeting mitochondrial factors for the development of antifungal therapies.

## 1. Introduction

Fungal infection is still an important medical issue worldwide and poses a significant threat to human health [1]. The severity of fungal infections and the concomitant importance of searching for new and better antifungal treatments are often underappreciated. The number of drugs available to treat fungal infections is limited, and those that are commonly used often suffer from being fungistatic rather than fungicidal [2,3]. Azoles, the first-line antifungals used in the clinic, are one of these fungistatic classes of drug [4,5,6]; they can decrease the production of ergosterol by inhibiting cytochrome P450 enzyme Erg11 and by damaging the cell membrane [7,8,9]. The fungistatic nature of the azoles coupled with their extensive use has resulted in azole resistance in populations of various pathogenic fungi [4,6,10,11]. Accordingly, there is an urgent need to unravel the molecular mechanisms of azole resistance to search for new and effective therapies.

Azole drug resistance and the fungal virulence are intimately intertwined with their metabolism, and mitochondria play a central role in that metabolism [12,13]. Mitochondria house and integrate multiple metabolic functions relating to lipids, iron metabolism, energy production, and cell wall biosynthesis [14,15,16,17,18,19], which are associated with fungal virulence and resistance to azoles. Mutations affecting mitochondrial functions including functioning of the electron transport chain (ETC), protein import, calcium homeostasis, mitochondrial genome maintenance, and mitochondrial transcription can result in avirulence and azole resistance or susceptibility [17,20,21,22,23,24,25]. The functions of mitochondria in these pathways are complex, and research findings open avenues for new, mitochondria-targeted therapeutic approaches. In this review, we describe the molecular mechanisms through which mitochondria are involved in regulating azole resistance and fungal pathogenicity and discuss the possibility for targeting mitochondrial factors for the development of antifungal therapies.

## 2. Role of Fungal Mitochondria in Azole Resistance

### 2.1. Calcium Signaling Participates in Mitochondria-Mediated Azole Resistance

**Figure 1:**
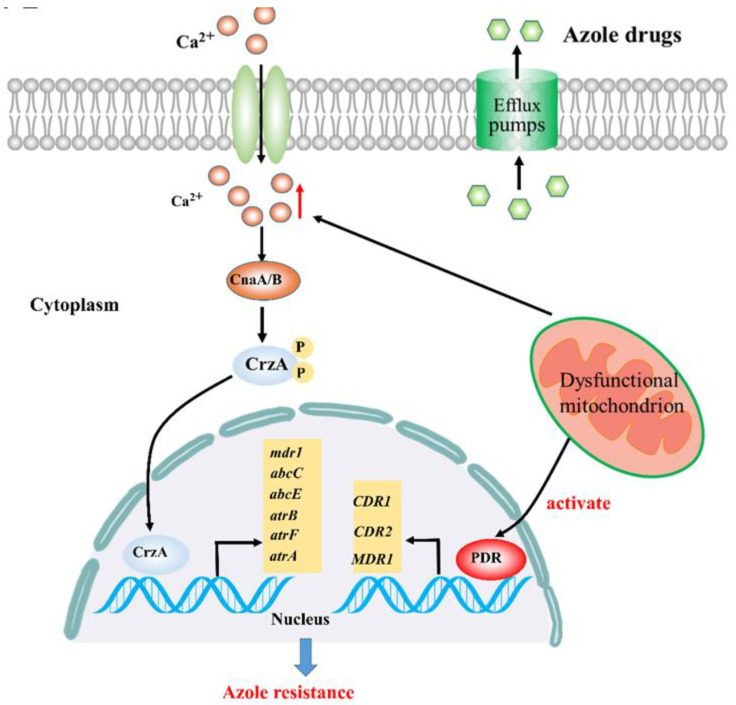
Calcium signaling participates in mitochondria-mediated azole resistance. Abnormal cytoplasmic Ca^2+^ transients induced by mitochondrial dysfunction cause a persistent nuclear localization of zinc finger transcription factor CrzA (*C. albicans* Crz1 homolog in *A. fumigatus*) and, therefore, triggers the global overexpression of multidrug transport genes (*mdr1*, *atrB*, *atrF*, *abcE*, *atrA*, and *abcC*), leading to azole resistance.

Mitochondrial dysfunction can initiate a mitochondrial–nuclear cross-talk that contributes to azole resistance in various pathogenic fungi [24,26]. For example, mitochondrial dysfunction caused by mitochondrial mutation can mediate azole resistance via activation of the pleiotropic drug resistance (Pdr) pathway that is involved in directly regulating the expression of drug efflux pumps (*CDR1*, *CDR2*, and *MDR1*) (Figure 1) [16]. Although this molecular mechanism has been well characterized, little is known about other molecular mechanisms underlying resistance to azoles arising from mitochondrial dysfunction. It has been reported that calcium signaling is involved in azole resistance in various fungi [27,28,29,30]. Defects in calcium homeostasis would lead to the retention of cytoplasmic calcium, which in turn activates calcineurin phosphatase activity [27]. The activated calcineurin will dephosphorylate zinc finger transcription factor Crz1, promoting nuclear translocation and induction of genes involved in azole resistance in *Candida albicans* [31]. Although the relationship between the calcineurin–Crz1 pathway and azole resistance has been determined, it is still unclear whether there is a direct relationship between the calcium signaling pathway and mitochondrial function. Recently, however, a study has established a close connection between mitochondrial function and calcium signaling-dependent azole resistance in *Aspergillus fumigatus* (Figure 1) [24]. Abnormal cytoplasmic Ca^2+^ transients induced by mitochondrial dysfunction cause a persistent nuclear localization of zinc finger transcription factor CrzA (*C. albicans* Crz1 homolog in *A. fumigatus*) and, therefore, triggers the global overexpression of multidrug transport genes (*mdr1*, *atrB*, *atrF*, *abcE*, *atrA*, and *abcC*) (Figure 1), leading to azole resistance [24]. Moreover, CrzA can directly regulate the gene expression of these multidrug transporters. Importantly, downregulation of CrzA markedly decreased the expression of the abovementioned multidrug transport genes [24], which suggested that it is indeed persistent nuclear localization of CrzA that determines the survival of strains with dysfunctional mitochondria in an azole-stress environment. Interestingly, in contrast to the persistent nuclear localization of CrzA induced by mitochondrial dysfunction in normal medium, supplementation of culture medium with calcium chelators strikingly prompted CrzA–GFP to localize in the cytoplasm, leading to significant restoration of azole drug susceptibility for *A. fumigatus* strains with dysfunctional mitochondria [24]; this indicates that it is indeed enhanced cytosolic Ca^2+^ transients caused by mitochondrial dysfunction that determine the localization of CrzA and hence azole susceptibility. Taken together, these data strongly suggest that defects in mitochondrial function might be a signal leading to activation of calcium signaling in response to azole stress. These findings suggest that disruption of calcium signaling may be a promising therapeutic strategy to fight against mitochondria-mediated azole drug resistance.

### 2.2. Linking Efflux Pump Activity and Mitochondrial Function in Azole Drug Resistance

**Figure 2:**
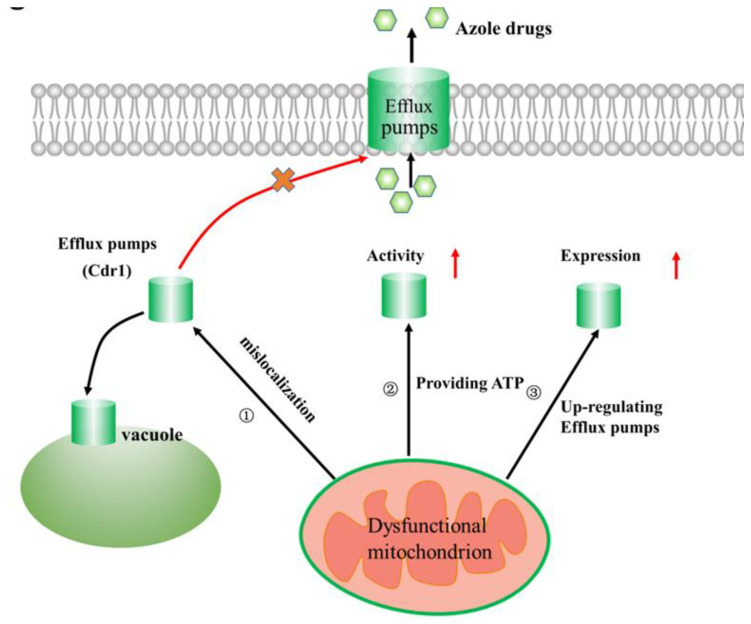
Linking efflux pump activity/expression and mitochondrial function in azole drug resistance: ① Dysfunctional mitochondria exert posttranslational regulation on Cdr1 activity by affecting its localization. The arrow means that mitochondrial dysfunction causes drug efflux pumps (Cdr1) to be located in vacuole rather than on cell membranes. ② Mitochondrial aerobic respiratory activity is increased in azole-resistant strains, and thus, the generation and conversion of ATP were increased in these strains with dysfunctional mitochondria, ensuring that drug efflux pumps gain more available ATP to increase the efflux of intracellular azole drugs. The arrow means that mitochondrial dysfunction leads to an increase in ATP content, which increases the activity of drug efflux pumps. ③ Mitochondrial fission defects lead to mitochondrial dysfunction, which in turn lead to a decrease in intracellular azole drug concentration through upregulating multiple efflux pumps, dampening the inhibition of lanosterol 14α demethylase (Cyp51A) by azole drugs. The arrow means that mitochondrial dysfunction leads to upregulate expression of multiple efflux pumps.

Although the role of drug efflux pumps in tolerance to azole drugs is well documented in pathogenic fungi [32,33], little is known about the molecular pathways involved in the regulation of drug efflux pumps. Generally, the regulation of drug efflux pumps includes gene expression regulation and activity regulation. As mentioned above, dysfunctional mitochondria exert translational regulation on efflux pump levels (Figure 1) [24]. Recently, a series of studies has also confirmed that dysfunctional mitochondria are involved in the regulation of efflux pump activity [14,34]. For example, localization studies on efflux pump Cdr1 revealed that the protein is largely missorted to the vacuole in *C. albicans* with dysfunctional mitochondria caused by deleting *Fzo1*, a key component required during biogenesis of functional mitochondria (Figure 2). As a consequence, there is reduced Cdr1 in the plasma membrane (PM), which contributes to decreased efflux activity of Cdr1 [14]. Recently, the antifungal potential of geraniol, a natural monoterpenoid from Palmarosa oil, against *C. albicans* was reported; it affects mitochondrial function. Mechanistic insights revealed that geraniol can work synergistically with fluconazole, specifically modulating Cdr1 activity. Confocal microscopy images depicted Cdr1 mislocalization in the presence of geraniol [34], indicating that reduced PM localization of Cdr1 as a result of its mis-sorting in the presence of geraniol is associated with increased susceptibility to azoles. Taken together, dysfunctional mitochondria may exert posttranslational regulation on Cdr1 activity by affecting its localization (Figure 2).

Mitochondria are the major cellular source of ATP, and the activity of efflux pumps is energy dependent [35,36]. Thus, there is a close relationship between mitochondrial aerobic respiration and the regulation of drug efflux pump activity. Recently, a series of studies has demonstrated that mitochondrial aerobic respiratory metabolism might be directly associated with efflux pump-mediated resistance of *C. albicans* to azoles [37,38]. Generally, mitochondrial aerobic respiratory activity is increased in azole-resistant *C. albicans* strains, and thus, the generation and conversion of ATP were increased in these strains [37]. Additionally, in these azole-resistant strains, the intracellular ATP content was also increased, implying that the transport of ATP from mitochondria to cytoplasm increased, ensuring that drug efflux pumps gain more available ATP to increase the efflux of intracellular azole drugs (Figure 2), revealing a molecular mechanism through which mitochondria affect azole resistance. Interestingly, the addition of tetrandrine, an inhibitor of the calcium channel blocker, to a culture of *C. albicans* could drastically decrease the intracellular ATP content, leading to the inhibition of drug efflux pump activity and thus resulting in increased azole susceptibility [38]. Taken together, mitochondrial aerobic respiratory metabolism may be directly involved in azole resistance mediated by azole efflux pumps. Of course, there is still much to learn about the role of mitochondrial aerobic respiration in azole resistance.

### 2.3. A Link Between Mitochondrial Dynamics and Azole Resistance

Mitochondria within cells continuously fuse and divide. This phenomenon is called mitochondrial dynamics, and it is crucial for mitochondrial function [26,39]. The fusion of mitochondria is mediated through Fzo1, which belongs to the GTPase family of proteins. As mentioned above, *Fzo1* mutants have mitochondrial fusion defects leading to mitochondrial dysfunction in *C. albicans*, which in turn causes increased susceptibility to azole drugs, mainly due to mis-sorting of Cdr1 efflux pumps to the vacuole [14]. In another fungal pathogen, *A. fumigatus*, mitochondrial fusion may not be involved in regulation of azole susceptibility. In contrast, mitochondrial fission, which depends on Dnm1, Mdv1, and Fis1, is important for azole susceptibility in *A. fumigatus*, and all three fission mutants showed a comparable increase in azole resistance [26]. Interestingly, simultaneous disruption of fusion and fission leads to even further increased azole resistance compared with the single fission mutant in *A. fumigatus*. Additionally, in *Candida glabrata*, mutations in *CgSHE9* encoding a protein that plays a role in mitochondrial fission lead to increased azole resistance [40], indicating that mitochondrial fission can play a general role in azole susceptibility. Although mitochondrial fission is related to increased azole resistance, the molecular mechanism underlying the increased azole resistance is still unclear. One possible mechanism in *A. fumigatus* is that mitochondrial fission defects lead to a decrease in intracellular azole drug concentration through upregulating multiple efflux pumps (Figure 2) [41], dampening the inhibition of lanosterol 14α demethylase (Cyp51A) by azole drugs. Recently, an important contribution of mitochondrial fission to azole susceptibility of *C. albicans* has been reported. The addition of dynasore, a mitochondrial fission inhibitor, to a culture of *C. albicans* could drastically increase azole susceptibility [42]. In an attempt to further understand how mitochondrial fission is associated with the response of *C. albicans* to azole drugs, proteomic analysis found decreased expression of proteins associated with mitochondrial structures and functions, the plasma membrane, and the cell wall in *C. albicans* with mitochondrial fission defects [42]. Of course, further research is required to understand the relationships between mitochondrial fission, azole susceptibility, and expression of these proteins.

### 2.4. Linking Fungal Lipid Biosynthesis and Mitochondrial Function in Azole Drug Resistance

**Figure 3:**
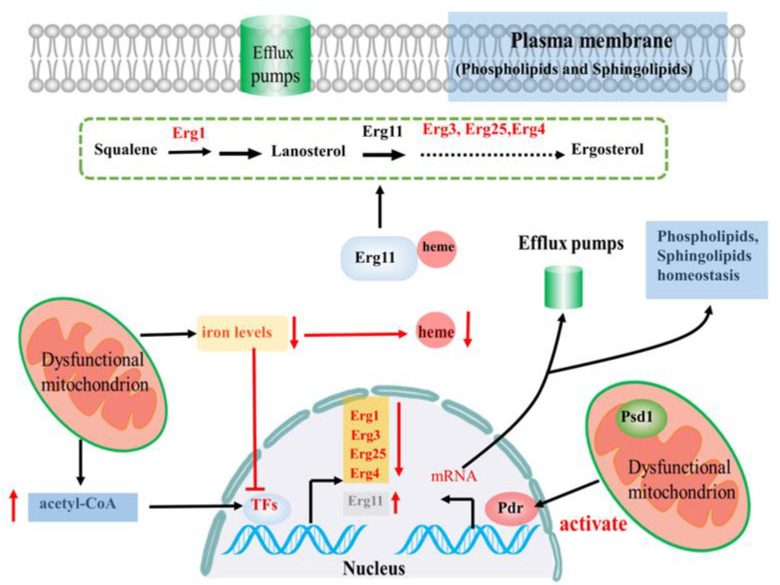
Linking fungal lipid biosynthesis and mitochondrial function in azole drug resistance: mitochondrial dysfunction leads to deregulation of iron metabolism and disturbs ergosterol biosynthesis, causing increased azole susceptibility. On the one hand, the disorder of iron metabolism leads to the deregulation of ergosterol biosynthesis gene expression (*Erg1, Erg3, Erg25*, and *Erg4*), with a commensurate decrease in ergosterol levels. On the other hand, the deregulation of iron metabolism also contributes to a decrease in ergosterol levels by decreasing heme levels. mitochondrial dysfunction due to mutated cofilin.

It is well known that azoles function as antifungal drugs by specifically targeting the fungal Erg11, preventing the biosynthesis of ergosterol [7,11]. Thus, the connection between ergosterol biosynthesis and azole resistance is strong. It has been reported that the absence of functional mitochondria can affect cellular iron homeostasis [18,43], which in turn regulates numerous cellular processes such as heme metabolism and ergosterol biosynthesis [7]; these are associated with azole drug resistance. Accordingly, there is a strong connection between mitochondrial function, cellular iron homeostasis, and ergosterol biosynthesis (Figure 3).

For instance, in *C. albicans*, mitochondrial dysfunction achieved by deleting *Fzo1*, a gene involved in mitochondrial fusion, leads to deregulation of iron metabolism and disturbs ergosterol biosynthesis, causing increased azole susceptibility [14]. On the one hand, the disorder of iron metabolism caused by deleting *Fzo1* leads to the deregulation of ergosterol biosynthesis gene expression (*Erg1, Erg3, Erg25*, and *Erg4*) (Figure 3), with a commensurate decrease in ergosterol levels. On the other hand, ERG11 and ERG5—key enzymes in ergosterol biosynthesis—are heme-containing enzymes, and iron is required for heme metabolism (Figure 3) [7]. Thus, the deregulation of iron metabolism in *ΔFzo1* mutants may also contribute to a decrease in ergosterol levels by decreasing heme levels, which eventually leads to increased azole susceptibility. Taken together, these data extend the link between mitochondrial function and iron homeostasis to an effect on ergosterol biosynthesis (Figure 3). Recently, a study has confirmed that mitochondrial dysfunction due to mutated cofilin affects ergosterol biosynthesis by regulating ergosterol biosynthesis gene *ERG11* expression in *A. fumigatus* [44]. The mutated cofilin triggered fatty acid β-oxidation, which increased acetyl coenzyme A (acetyl-CoA) and ATP concentrations. The extra ATP supplies provided additional energy to drug efflux pumps, while excess acetyl-CoA induces overexpression of *ERG11* gene (Figure 3), affecting the ergosterol biosynthetic pathway and thus reducing susceptibility to azoles [44]. Taken together, the connection between mitochondrial function, ergosterol biosynthesis, and azole drug resistance is strong, but the mechanistic details are far from understood.

Many studies have also confirmed that mitochondrial dysfunction can lead to a change in lipid structure and the composition of mitochondrial membranes, which may lead to azole resistance [16]. It has reported that the changes in the mitochondrial membrane would lead to diminished membrane association of phosphatidylserine decarboxylase Psd1 in the mitochondrial inner membrane, which in turn leads to activation of the Pdr pathway (Figure 3) [45]. On the one hand, activation of the Pdr pathway leads to increased expression of genes encoding drug efflux pump proteins [46,47,48,49]. On the other hand, activation of the Pdr pathway regulates the expression of genes required for the homeostasis of two key lipid components of plasma membranes, phospholipids, and sphingolipids (Figure 3) [15,50]. Because plasma membrane lipid components are crucial for surface localization of drug efflux pumps [50,51], the Pdr pathway may provide coordinate control of plasma membrane lipid composition through modulation of sphingolipid and phospholipid homeostasis and of drug efflux pump proteins that function in the resulting membrane lipid environment, which may contribute to Pdr-mediated azole resistance upon mitochondrial dysfunction. Taken together, mitochondrial function plays an important role in membrane lipid composition, and activation of lipid homeostasis by the Pdr pathway may serve to compensate for the changes to membrane lipid structure and composition upon mitochondrial dysfunction, which may be the reason why fungi activate the Pdr pathway upon mitochondrial dysfunction.

## 3. Roles of Mitochondria in Fungal Pathogenicity

### 3.1. Mitochondrial Morphology Influences Fungal Pathogenicity

**Figure 4:**
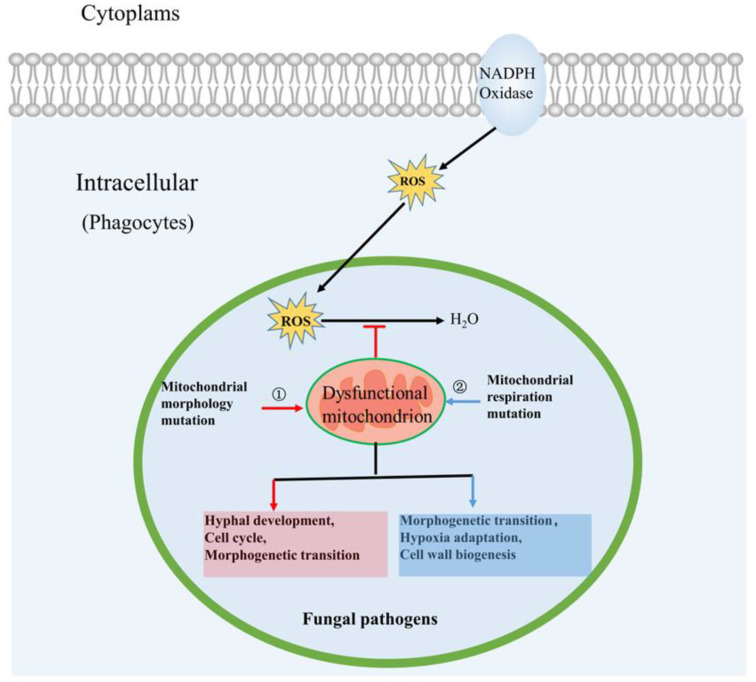
Roles of mitochondria in fungal pathogenicity: ① Mitochondrial morphology influences fungal pathogenicity. ② Mitochondrial respiration influences fungal pathogenicity.

Mitochondria are dynamic organelles that constantly undergo fusion and fission to maintain mitochondrial function during healthy growth and in stress conditions [52,53]. Recently, the fusion and fission of mitochondria have emerged as important contributors to the fungal virulence in a variety of fungal pathogens [52,54]. Furthermore, molecular mechanisms by which mitochondrial morphology affects fungal virulence have been elaborated in detail (Figure 4). In this section, we will focus on the role of mitochondrial morphology in fungal virulence.

The fusion of mitochondria can help cells cope with various stress conditions by forming what is termed tubular morphology that can share genetic material and resources to aid survival [52,55]. In *Saccharomyces cerevisiae*, fusion of mitochondria is mediated by three dynamin-related proteins (DRPs), Fzo1, Mgm1, and Ugo1 [26,52,54], which are conserved and have been characterized in fungal pathogens. For example, in *C. albicans*, deletion of the mitofusin Fzo1 results in decreased ability to adopt tubular morphology, which in turn decreases the ability of cells to resist peroxide stress that is encountered during infection of a host by the fungus (Figure 4) [54]. Mgm1 has an important role in the regulation of mitochondrial fusion, mtDNA (i.e., mitochondrial genome) maintenance, and hyphal development in *C. albicans*. Fungal cells lacking Mgm1 cannot progress through the cell cycle due to mitochondrial fusion defects and mtDNA loss, revealing a molecular mechanism through which Mgm1 affects *C. albicans* virulence in hosts [56]. Because the morphogenetic transition is thought to be related to *C. albicans* virulence, the deficiency of hyphal development caused by deletion of *Mgm1* may be another reason for attenuation of virulence in a mouse model of disseminated infection. In another fungal pathogen, *A. fumigatus*, mitochondrial fusion, which depends on Mgm1, Ugo1, and Fzo1, is essential for mtDNA maintenance and viability [26]. When examined for virulence, a strain of *A. fumigatus* with a conditional mutation in the essential gene *Mgm1* was avirulent in the *Galleria mellonella* infection model. Similar to *C. albicans* and *A. fumigatus*, recent research has found that mitochondrial fusion is also involved in *Cryptococcus neoformans* virulence in hosts, implying that mitochondrial fusion may play a general role in fungal virulence [54]. *C. neoformans* is a facultative intracellular pathogen that is exposed to oxidative stress within host cells. Thus, *C. neoformans* must be able to resist oxidative stress in phagocytes to survive [57]. Fzo1 plays an important role in mitochondrial fusion in *C. neoformans*, and a *∆fzo1* mutant is severely defective in growth in the presence of hydrogen peroxide (mimicking the host intracellular environment). Interestingly, supplementation of culture medium with reactive oxygen species (ROS) scavengers could reverse the growth defect of the *∆fzo1* mutant within phagocytes, which shows that failure of the *∆fzo1* mutant to clear ROS efficiently is the main reason for its inability to survive within phagocytes [54]. Taken together, a marked reduction in growth in the presence of hydrogen peroxide determines the failure of the *∆fzo1* mutant to survive within phagocytes, making the *∆fzo1* mutant avirulent in a macrophage cell line.

Mitochondrial fission plays a lesser role than mitochondrial fusion in fungal pathogenicity [35]. In fungi, the fission of mitochondria is mediated by three DRPs, Dnm1, Fis1, and Mdv1, which are conserved and have been characterized in fungal pathogens [35]. A series of studies has demonstrated that inactivation of mitochondrial fission has a bigger effect on cellular growth in filamentous fungi than in yeasts. For example, in the yeasts *C. albicans* and *C. neoformans*, mitochondrial fission mutants displayed normal growth phenotypes [54]. In contrast, in the filamentous pathogen *A. fumigatus*, mitochondrial fission mutants showed drastically reduced hyphal growth in vitro and aberrant mitochondrial morphology compared with wild-type strains [26]. However, these mitochondrial fission mutants displayed normal virulence in a *G. mellonella* infection model, suggesting that mitochondrial fission may not be essential for the virulence of *A. fumigatus*.

### 3.2. Mitochondrial Respiration Influences Fungal Pathogenicity

To colonize, infect, and invade a host to cause disease, fungal pathogens must adapt their metabolism and generate energy for adaptation to the host environment [12,58,59,60]. Most fungal pathogens use oxidative respiration for energy generation, which is the most efficient pathway to produce ATP [20,35]. There is a close link between mitochondrial respiration and fungal virulence. Mitochondrial respiration deficiency leads to attenuated virulence in *C. albicans* and *A. fumigatus* [21,60,61,62,63]. Moreover, relevant molecular mechanisms have been well studied (Figure 4). On the one hand, mitochondrial respiration can affect the morphogenesis of fungi. For instance, high ATP levels resulting from mitochondrial respiratory activity have been shown to be crucial for *C. albicans* to switch from yeast cells to mycelium growth [20,64,65]. Because this morphogenetic transition is thought to be related to *C. albicans* virulence [66], this result shows that mitochondrial respiration can mediate *C. albicans* virulence by affecting morphogenetic transition. On the other hand, mitochondrial respiration plays an important role in hypoxia signaling and adaptation [60]. Hypoxia is an important component of host microenvironments during fungal infections. Thus, adaptation to hypoxia might be a critical factor in the ability of fungal pathogens to cause lethal disease [60]. It has been reported that mitochondrial respiration is active in hypoxic conditions and that components of the electron transport chain (ETC) are involved in mediating resistance to oxidative stress encountered by *A. fumigatus* during infection. A cytochrome *c* null-mutant of *A. fumigatus* that lacked mitochondrial respiratory function was significantly attenuated in virulence [60]. Taken together, mitochondrial respiratory plays an important role in fungal pathogenicity, highlighting the need for more mechanistic studies on this topic.

The structure and composition of the cell wall determine the recognition of pathogenic fungi by the host’s immune system [67,68,69]. Accordingly, cell wall biogenesis and integrity are associated with fungal pathogenicity. Recently, mitochondrial respiration has been proved to be important for cell wall biogenesis and integrity [70]. Fungal mutants lacking mitochondrial respiration tend to exhibit hypersensitivity to cell wall-targeting drugs, implying that their cell wall structure and organization have been changed [70]. In *C. albicans*, some results have confirmed that *Goa1*, *Nuo1*, *Nuo2*, and *Ndh51*, genes involved in regulation of mitochondrial respiration, play an important role in cell wall integrity and pathogenicity [21,71,72]. Inactivation of *Goa1*, *Nuo1*, or *Nuo2* leads to decreased mitochondrial respiratory function, and loss of any one of these genes results in increased sensitivity to cell wall-targeting drugs and increased ROS production [21,70]. On the one hand, ROS as signal molecules can regulate cell wall biosynthesis. On the other hand, ROS induced by respiratory blocks could lead to lipid homeostasis defects, which may underlie the cell wall phenotypes observed in mitochondrial respiration mutants more broadly [70,73]. These findings imply that there is a strong connection between mitochondrial respiration, ROS, cell wall biosynthesis/integrity, and lipid homeostasis, with consequent effects on fungal pathogenicity.

## 4. Potential for Mitochondrial Factors as Novel Antifungal Therapeutic Targets

The classic antifungal drugs used to treat fungal pathogens do not rapidly inhibit fungal growth, and hence, mortality rates remain unacceptably high. To counter these problems, the development of new therapeutic approaches is essential. As described above, fungal pathogens require mitochondrial function for normal growth, azole drug resistance, and virulence. Given the central role of mitochondria in processes essential for adaptability, growth, and maintenance, coupled with the presence of fungal-specific characteristics, it may be possible to develop therapies based on inhibition of fungal mitochondria.

As described above, Fzo1 is a major player in mitochondria-related azole resistance and virulence [14]. Although Fzo1 proteins are highly conserved in fungi, plants, and animals, they have specific characteristics in fungi. Notably, the *C*-terminal region of Fzo1 proteins in animals and fungi has no detectable sequence similarity and cannot be reliably aligned. Bioinformatic predictions showed that fungal Fzo1 proteins carry two predicted *C*-terminal TMDs whereas Fzo1 proteins in animals have only a single predicted *C*-terminal TMD [74]. Thus, any compound inhibiting the function of fungal Fzo1 by targeting its *C*-terminal TMD without affecting the human homolog would be theoretically valid. Taken together, these data indicate that these specific *C*-terminal TMD of Fzo1 may serve as targets for developing novel antifungal therapies.

The mitochondrial respiratory pathway is an effective target for fungicides to control fungal growth. Additionally, the presence of fungal-specific respiratory components and the recent discovery of the association between respiration and pathogenesis in several fungal pathogens have promoted the development of new mitochondria-targeted fungicides [20]. Recently, a study identified seven genes (*Nuo3*, *Nuo4*, *Nue1*, *Nue2*, *Qce1*, *Coe1*, and *Coe2*) that are unique to the CTG fungal clade, which is so named because they generally translate CTG as serine rather than leucine. The CTG fungal clade contains multiple important human pathogens, including *C. albicans*, and showed that they are required for full mitochondrial respiratory metabolism and fungal virulence [13], implying that these clade-specific mitochondrial factors might represent novel antifungal therapeutic targets. The mitochondrial respiratory chain is composed of four large multi-subunit enzymes, complexes I to IV. Of these, complex I has the highest molecular weight and, energetically speaking, is responsible for generation of approximately half of the ATP [35]. Complex I is present in most fungal pathogens, and recent work has identified two subunits (Nuo1 and Nuo2) of complex I itself as well as the complex I regulator Goa1 to be fungus-specific [21,75]. Loss of these proteins can lead to deficiencies in respiration and virulence [21,72]. Similarly, deletion of *Nue1*, *Nue2*, *Nuo3*, or *Nuo4* can impair complex I function, causing deficiencies in respiration and virulence [13], making them attractive antifungal drug targets. Additionally, dysfunction of complex I can lead to ROS accumulation in mitochondria, which in turn promotes fungal cell death [76]. Thus, inhibitors of complex I have fungicidal activity by increasing mitochondrial ROS levels. Taken together, fungal-specific mitochondrial factors regulating the function of complex I may represent novel antifungal therapeutic targets.

In addition to the classical ETC, many fungal pathogens possess a cyanide-insensitive alternative oxidase (AOX), which can permit respiration when the classical electron transport chain is inhibited, thus maintaining growth and viability [77,78]. As AOX is absent from mammals, it has been investigated as a potential antifungal target [79]. Because AOX is dispensable for virulence in some fungal pathogens [60], AOX inhibitors as antifungal agents may not be universally successful, at least not as monotherapy. Therefore, a combination of alternative respiration and classical pathway inhibitors may be the most effective antifungal strategy. For example, a combination of the AOX inhibitor salicylhydroxamic acid (SHAM) and fluconazole displayed synergistic antifungal activity against *C. albicans* [80]. The only AOX protein structure available is of that from the human parasite *Trypanosoma brucei* (a kinetoplastid, not a fungus) [81]. On the basis of AOX structural information, some new inhibitors, such as ascofuranone, have been discovered [81]. However, because of the lack of structural information about fungal AOX, the development of fungal AOX inhibitors has been hampered. Recently, novel fungal AOX inhibitors, optimized *N*-phenylbenzamide derivatives, were shown to effectively inhibit spore germination of the phytopathogen *Moniliophthora perniciosa* [82]. Of course, it is necessary to further study the structure and physiological activity of AOX in fungi and the structure–activity relationships of existing AOX inhibitors, which will promote the development of effective fungal AOX inhibitors to control fungal reproduction.

During the infection process, fungal pathogens are challenged by massive changes of the environmental conditions, e.g., by nutrient depletion, elevated temperatures, and hypoxia. Oxygen availability drops from 21% in the atmosphere to less than 1% in inflammatory and necrotic tissue [83]. Thus, hypoxic microenvironments were often found to exist at the site of infection in mice infected with fungal pathogens, and the adaptation to hypoxia might be a critical factor in the ability of fungal pathogens to cause lethal disease. Currently, a series of studies have confirmed that functional mitochondria play an essential role in the adaptation process towards hypoxia of several pathogenic fungi [83]. The mitochondrial aerobic respiration is active during hypoxia and the protein levels of all respiratory complexes also increased under hypoxic growth conditions to increase the respiratory capacity of mitochondria [83]. Recently, proteome analysis identified a mitochondrial protein HorA to be highly upregulated in *A. fumigatus* during hypoxic adaptation [83]. HorA is associated with biosynthesis of coenzyme Q, which is involved in mitochondrial respiration and maintenance of cellular redox homeostasis. Therefore, the loss of HorA displayed an impaired response to both oxidative and reductive stress and showed significantly attenuated virulence. Moreover, an increased resistance against azole drugs was also observed. Taken together, HorA plays a critical role in the virulence of *A. fumigatus*. Noteworthily, due to its absence in mammals, the HorA may represent a promising target for the development of novel antifungal drugs.

As described above, mitochondrial function is associated with ergosterol biosynthesis, indicating that mitochondrion inhibitors have the potential to enhance the effects of current azole drugs that target ergosterol biosynthesis. For example, inhibition of mitochondrial aerobic respiration with tetrandrine can cause increased susceptibility to azole drugs [38], fungal-specific inhibitors of complex III can reverse azole resistance [17], and a combination of the AOX inhibitor SHAM and fluconazole displayed synergistic antifungal activity [80]. Taken together, because of the connection of mitochondria to ergosterol metabolism, mitochondrial inhibitors may prove to be effective against fungal pathogens in combination with current azole drugs. It has been reported that the mitochondrial outer membrane Sorting and Assembly Machinery (SAM) complexes Sam35 and Sam37 are required for mitochondrial biogenesis and dynamics. Sam35 is required for growth and virulence of *C. albicans*, and Sam37 is critical for cell wall integrity and virulence. Importantly, there are significant structural differences in fungal Sam35 and Sam37 compared with their animal counterparts [23], and thus, they could be explored as targets for antifungal drug development. Future research will focus on screening and developing small molecule compounds that can inhibit these proteins.

## 5. Conclusions and Future Prospects

Because of the connections of mitochondria to azole resistance, lipid metabolism, pathogenesis, and cell wall regulation, pharmacological disruption of mitochondrial function may prove to be effective against fungal pathogens. Combining a mitochondrial function inhibitor with one or more current antifungals (azole, polyene, and echinocandin) could increase efficacy, reduce toxicity, and prevent the emergence of antifungal drugs resistance better than monotherapy regimens. Additionally, fungal pathogens must be able to resist oxidative stress in phagocytes to survive, and mitochondrial function plays an important role in oxidative stress resistance. Therefore, the pharmacological damage of mitochondrial function will lead to impaired capacity to cope with the oxidative stress from host cells and inhibit the growth of fungal pathogens in the host. Taken together, the regulation of mitochondrial function provides promising therapeutic targets for combating fungal infection. Furthermore, by targeting mitochondrial function with a specific combination of two antifungal drugs, the possibility that fungal pathogens will develop mutations that increase azole drug resistance is significantly lessened. Therefore, we hope that future efforts will focus on finding new compounds that specifically block mitochondrial function. An important challenge for future mitochondria-targeting therapy is developing selectivity for mitochondrial factors. In this regard, structural analysis of mitochondrial factors would be an important next step to help guide medicinal chemistry efforts. Although some studies have emphasized the importance of mitochondrial function for azole resistance and fungal virulence, the exact molecular mechanisms are not fully understood. Thus, it is important to understand the underlying mechanisms of mitochondria-mediated azole drug resistance and fungal pathogenicity. Taken together, it is worthwhile to study how mitochondria promote azole drug resistance and fungal pathogenicity.
